# Development of Clause Chaining in Korean

**DOI:** 10.3389/fpsyg.2020.00256

**Published:** 2020-03-11

**Authors:** Soonja Choi

**Affiliations:** Department of Linguistics and Asian/Middle Eastern Languages, San Diego State University, San Diego, CA, United States

**Keywords:** Korean acquisition, clause chaining in Korean, topic continuity, anaphoric reference, acquisition of conjunction, event segmentation

## Abstract

Korean is a language with the verb at the end of a clause/sentence. In chaining several clauses [each consisting of a subject and a verb] in a sentence, a conjunction (e.g., *-ko* “and then,” *-ese* “because, and so”) is suffixed to the verb of a non-final clause. Korean has an extensive set of conjunctions that connect to the next clause, expressing temporal, causal, and contrastive relations among others. In this paper, I lay out a developmental trajectory of clause chaining construction in Korean based on longitudinal and cross-sectional data samples, focusing on conjunctive forms and functions as well as morphological and syntactic properties of connected clauses in a sentence. The database comes from longitudinal naturalistic speech data of five children collected regularly over different time periods between 2 and 5 years of age, and from elicited descriptions of short video events from children - aged 3, 4, 6, 8, and 10 years - and adults. The results show that, at least from 2 years of age, the Korean children in the sample start connecting clauses using appropriate conjunctions. Within 6 months, they acquire several major conjunctions that express temporal, causal, conditional, and contrastive relations between events. By 4 years of age, the children’s clause chains are quite adult-like in terms of the repertoire of conjunctive forms and functions, and of the morphological and syntactic features of the clauses that connect to the main clause. In particular they learn to express temporal relations that have some disjuncture between events. However, 4-year-olds still lack the ability to appropriately refer to differential subjects of the chained clauses and also to connect multiple clauses in a sentence. The elicitation data reveal that further development in clause chaining occurs over several years – with a milestone at 10 years – and through adulthood, particularly in relation to appropriate referential marking, conjunction frequency, and segmentation of a macro event into sub-events for clause-chaining construction. These developmental processes are presented from a cognitive perspective, in particular with regard to concept of temporal relation, reference specification involving two or more entities, and perceptual saliency of event type.

## Introduction

Korean is a verb-final (SOV) language with rich agglutinative morphology. Grammatical markers, such as case markers and verb inflections, are suffixed to the corresponding nouns and verbs, respectively, with clear boundaries, as in the following examples:


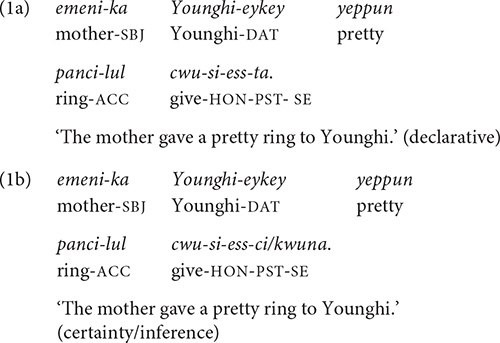


As shown in (1a,b), the subject marker -*ka(/-i)* and the indirect object marker *-eykey* are suffixed to the corresponding nouns, and the verbal inflections, e.g., the honorific marker *-si-* referring to *emeni* “mother” and the past tense marker -*ess(/ass)-*, are suffixed to the verb stem, *cwu-* “give.” In Korean, a sentence always ends with a SE suffix. In written reports and formal speeches, the SE suffix is typically the neutral declarative ending *-ta*^1^ (1a). But in spoken narratives and conversations, a variety of SE suffixes are used, such as *-ci* and *-kwuna* in (1b). These SE suffixes typically carry epistemic/evidential meanings: They express the speaker’s assessment about the truth of the proposition (i.e., degree of certainty) or the type of evidentiality (e.g., inference, hearsay) (Lee, 1991; Choi, 1995; Sohn, 2018). The SE markers in spoken discourse are highly interactional as they convey not only the speaker’s assessment of an event/state, but also incorporate his/her understanding of the listener’s knowledge about it. For example, the SE suffix *-ci* in (1b) means not only that the speaker is quite certain about the truth of the event but also that he/she believes that the listener shares the certainty of the event. (The SE markers can be followed by a speech register marker, for example, *-yo*, which marks politeness to the addressee).

As a head-final language, modifiers in Korean - whether they are phrasal or clausal - precede their heads. Thus, in (1a,b), the adjective “pretty” precedes the noun “ring.” Similarly, in a complex sentence, a subordinate clause precedes its head, which could be a noun phrase, a verb phrase, or the main clause. Thus, in (2), the relative clause “the mother gave to Younghi” precedes its head noun, *kes* “thing”, and in (3), the object complement clause “that the mother gave (her) the ring” precedes the verb phrase *malhay-ess-ta* “say-PST-SE.” (The subject of the main clause, *Younghi-ka* “Younghi-SBJ,” can be separated from its verb occurring at the beginning of the sentence, i.e., before the object complement clause).


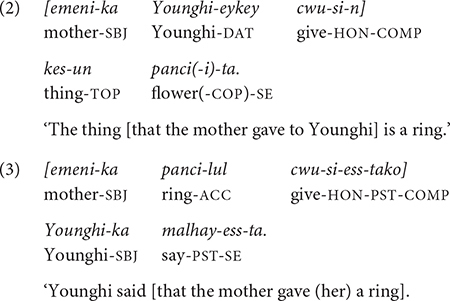


The same head-final rule applies when the complex sentence involves clause chaining, which consists of one or more non-main (and non-final) adverbial clauses and a main (final) clause. In clause chaining, which is the topic of the present study, speakers provide additional information (e.g., time and cause) to the main event using adverbial clauses. In Korean, an adverbial clause canonically precedes the main clause. For example, in (4), the event of “ball hitting the vase” in the first clause explains the cause of the “vase breaking” in the second clause, which is the main clause. The causal relation between the two clauses is signaled by the conjunction *-ese* “because/as,” which is suffixed to the bare stem of the medial verb *chi-* “hit.”


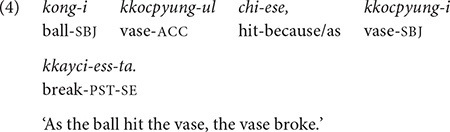


The present study examines how Korean children acquire the clause-chaining construction. In particular, I investigate some morphological and semantic aspects of the ways in which children acquire conjunctions and other grammatical features of clause chaining. I examine the development from 2 years of age and onward, using both longitudinal spontaneous speech data and cross-sectional elicitation data. In the following section, I first lay out the characteristics of the construction that are relevant to the present study.

### Characteristics of the Clause Chaining Construction in Korean

In clause chaining, one or more adverbial clause(s) is/are connected to the main clause, providing information about the time, cause, manner, condition, or circumstance of the main event (see the section “Conjunctive Suffixes and Their Functions”). Adverbial clauses canonically occur in a medial/non-final position of the sentence, and the main event is typically, but not always, the final clause of the sentence. In this paper, I will refer to these non-final adverbial clauses as “medial” clauses, although they may not always adhere to the established definition of a “medial clause” in that in Korean the verbs of “medial clauses” can be specified for tense (e.g., past tense, *-ess*) (see the section “Non-finiteness/Finiteness of the Verb/Predicate of a Medial Clause”). As in many other clause chaining languages, each adverbial clause ends with a verb and a conjunctive suffix, which connect both morphologically and semantically to the immediately following clause (Longacre, 1985; Watanabe, 1994; Dixon, 2009). These characteristics are illustrated in the following example, taken from the database of the present study (see the section “Cross-sectional Elicitation Study”):


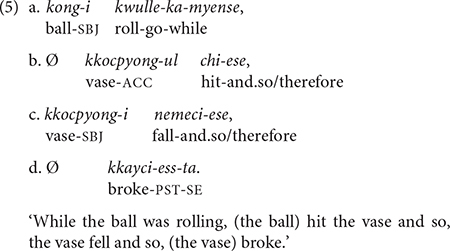


Example (5) has three medial clauses (5a–c), each ending with a conjunction that connects to the next clause with a specific adverbial meaning. The first clause (5a) specifies the manner of the ball’s motion (i.e., rolling) and provides the temporal frame of the ball hitting the vase (5b) with its conjunctive suffix *-myense* “while.” Clauses (5b) and (5c) describe the series of events that led to the main event of the vase breaking (5d).

#### Conjunctive Suffixes and Their Functions

There are many conjunctive suffixes in Korean. Sohn (2009) lists about a hundred different forms that express specific semantic notions relating to time, cause, manner, condition, purpose, contrast, and so on. However, the list of commonly used conjunctions in spoken Korean is much shorter. Kim (1992), who examined clause chaining in adult narratives [elicited from 10 participants using the “pear” film clip (Chafe, 1980)], found a total of 15 conjunction types in her data. Of these conjunctions, Kim (1992) analyzes and reports on only six forms, which have a level of frequency – 20 or more tokens in the entire database – that warrant detailed investigation: *-ko* “and,” *-ese* “and.then,” *nuntey* “given.that,” *-nikka* “because,” *-myense* “while,” *-taka* “while doing.” (These six conjunctions are marked with asterisks in Table 1).^2^ I should further note that, among these conjunctions, the form *-ko* “and” is reported to be the most frequent and thus is the most-studied conjunctive in clause chaining in Korean adult grammar (e.g., Lee, 2003; Kwon and Polinsky, 2005; Jendraschek and Shin, 2018). In the present study, the children in the longitudinal study use a total of 27 different connective forms between 2 and 5 years of age in their spontaneous speech, of which 12 suffixes are more frequently produced, with *-ko* “and” at the top of the frequency list (see the section “Development of Conjunctive Forms and Functions”).^3^ The 12 suffixes are listed in Table 1 with examples from the database of the present study.

**TABLE 1:** Conjunctive forms and functions with examples from children’s spontaneous speech data.

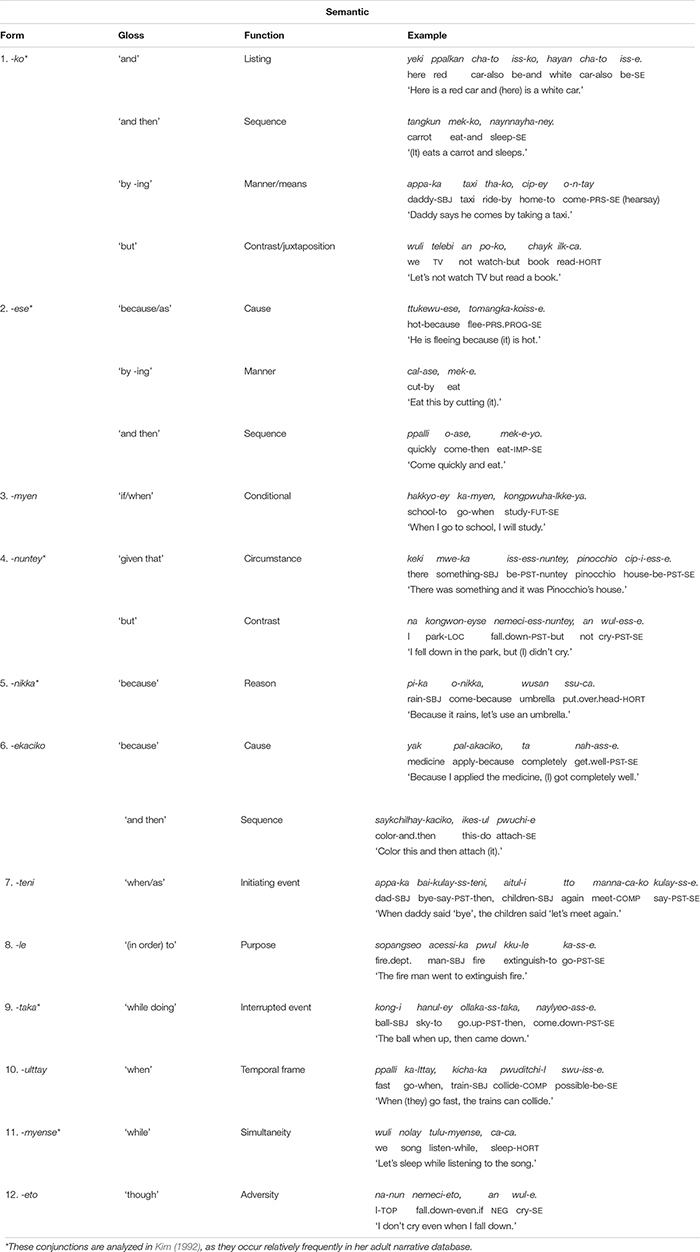			

The relationship between conjunctive forms and their functions is quite complex in Korean. On the one hand, the same form can serve several functions. For example, the most frequent conjunction, *-ko*, serves temporal, causal, and contrastive functions as well as a listing function (Table 1). Conversely, the same function can be expressed by several forms. For example, the temporal sequence function, “and then,” can be expressed by *-ko*, *-ese*, and also by *-ekaciko*, as shown in Table 1. However, in many cases, such overlap between forms and functions is only at a general level. Detailed analyses of the way in which a given conjunction semantically relates to the next clause suggest that each form typically serves a specific function that is subtly but importantly different from the similar functions served by other forms (Sohn, 2009; see the section “Early-Acquired Conjunctive Forms and Functions”).

#### Topic Continuity and Subject Marking

Topic continuity in clause chaining relates to the continuation (or discontinuation) of the nominal reference – particularly regarding the subject – from one clause to the next. Two aspects of topic continuity are worth noting regarding Korean. First, unlike some clause chaining languages (e.g., many Papuan languages), Korean does not have a switch-reference marker that explicitly signals whether the subject in the immediately following clause will have the same or a different subject from the current clause. However, in many cases, a given conjunction has a clear preference for the same subject (SS) or a different subject (DS) in the next clause. To this end, Korean conjunctive forms have a topic continuity feature (Kim, 1992). For example, the conjunction -*myense* “while” in (5a) is almost always followed by SS in the next clause, whereas the conjunction *-ese* “because/and.so” with a causal function often allows DS, as in (5b). In the present study, we will examine the topic continuity feature in children’s and adults’ use of Korean conjunctions.

Second, the major referential tools in Korean are full NP and zero anaphora. Korean has a system of pronouns, but it is infrequently used in conversations and narratives (Kim, 1992). A full NP is used when introducing a new character in the discourse or when the reference needs to be clarified, such as when two or more characters have been mentioned. When the referent is clear from the context or preceding discourse, arguments can be omitted. In clause (5b) above, the subject is omitted (=zero anaphora), which signals that it is the same, *kong* “ball,” as the subject of the preceding clause (5a). (The same is true for 5c,d).

Zero anaphora is a communicatively efficient tool when the listener can retrieve the correct referent from the context or prior discourse. On the other hand, it can also cause communicative breakdown when the referent is not recoverable, for example, when the speaker actually had not mentioned the referent earlier or the referent is not recoverable due to the distance between the prior mention and the ellipsis or because there are several possible candidates for the referent (Clancy, 1992). In the present study, we will examine how efficient young children at different ages are with their referential choices (full NP or zero anaphora).

#### Non-finiteness/Finiteness of the Verb/Predicate of a Medial Clause

In clause chaining, the verb/predicate of a medial clause is typically non-finite, meaning that the verb is in its bare stem and a conjunction is directly suffixed to it. The verb’s tense/aspect/modal information is accessed from the fully inflected verb in the main clause, as in (4). In Korean, however, in some cases, the verb of a medial clause is inflected. Kim (1992) notes that the finiteness of the verb depends on “the degree of grammatical or conceptual integration in the discourse” (p. 122). Such characterization explains the Korean system well. For example, verbs of an adverbial/medial clause with the conjunction -*myense* “while” are always non-finite as the conjunction denotes the overlap (i.e., integration) of a non-main event with the main event. On the other hand, verbs with conjunction *-nuntey* “but” can be finite, as the two events are juxtaposed, i.e., non-integrated.

#### Event Segmentation for Clause Chaining

Events can temporally overlap – fully or partially – or occur sequentially without clear perceptual boundaries between them. Take, for example, the ball-vase event sequence described in (5a–d): First, a ball rolls forward and hits a vase which, as a result, tips over and then breaks (see details in the section “Materials and Design”). These events flow from one to the next without pauses (i.e., no boundaries).

In order to linguistically describe the sequence of these events as one occurring before another or one causing the next, one needs to first segment connected events into event units (Baldwin et al., 2001, 2008; Levine et al., 2019). Segmented units can then be expressed as separate clauses and be connected with specification of the semantic relation between them. From the acquisition point of view, this process raises interesting questions: How do children segment event sequences for the purpose of linguistically encoding them? From a series of connected events, which types of event stand out to children for linguistic expression? In the present study, we will address these questions systematically with an experimental elicitation database.

#### Constructions Excluded From Analysis: Serial Verb Constructions and Clauses With Conjunctive Adverbs

By definition, clause chains are multi-clausal, each medial clause having its own subject and predicate that connect to the next clause with a particular adverbial meaning. In this section, I clarify that two types of construction – serial verb constructions (SVCs) and clauses headed by conjunctive adverbs – will be excluded from the present analysis as they do not fit this definition. First, SVCs are excluded as they are typically considered mono-clausal (e.g., Lee, 1992; Sohn, 2009; Chae, 2015). In the SVC, two or more verbs are strung together with the connective *-e*, as in (6). These verbs form a verb phrase with no intervening material (e.g., nouns and adverbs) between them. Basically, it is a single predicate consisting of a sequence of verbs (Aikhenvald, 2006). The verbs share the same subject argument and express different aspects of a single event (e.g., Lee, 1992; Chung, 1993; Sohn, 2009; Chae, 2015), in particular, a single motion event (Zubizarreta and Oh, 2007; Pyoun, 2011). For example, in (6), the two verbs, *nayli-* “descend” and *ka-* “go” describe the path (“descending”) and the deictic (“going”) aspects of the single motion performed by John (Choi, 2016). Whereas the final verb carries tense/aspect/modality inflections, non-final verbs are in bare stem forms.

In addition, unlike conjunctions such as *-ko* “and then” and *-ese* “because,” the connective *-e* does not have semantic content – it does not imply coordination or subordination – and simply serves to connect one verb stem to the next (Lee, 1992; Chung, 1993). Based on this analysis, the SVC, as characterized here, will be excluded from the present study on clause chaining.^4^


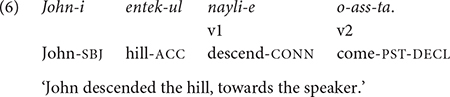


The present study also excludes sentences with free-standing conjunctive adverbs, which typically occur at the beginning of a sentence and which semantically connect to the preceding sentence (Sohn, 2009). For example, the conjunctive suffix *-ko* in (7) has a corresponding free-standing adverb, *kuli-ko*, as in (8). *Kuli-* in (8) indexes the event of the preceding sentence. I only examine complex sentences involving non-main clauses connected to the main clause by a conjunctive suffix.


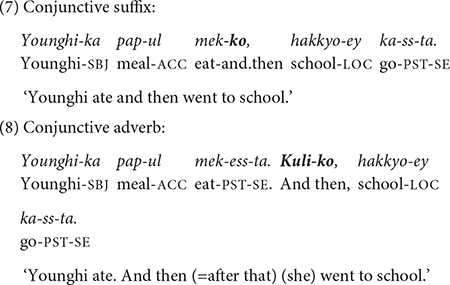


Examples (7) and (8) convey the same information, but they differ in syntactic structure. In (7), the two clauses, Younghi eating and (Younghi) going to school, are connected and form a complex sentence with a conjunctive suffix at the end of the medial clause. But in (8), each clause stands alone as an independent sentence: The semantic relation of the two sentences is expressed by the conjunctive adverb in the second sentence. In this paper, I will only be concerned with complex clause chaining sentences such as (7).

### The Present Study: Goals, Scope, and Database

Although there are several notable studies on clause chaining in the adult grammar in Korean (e.g., Kim, 1992; Lee, 2003; Kwon and Polinsky, 2005; Sohn, 2009; Chae, 2015), few studies have been conducted on how children acquire the Korean clause chaining construction. Therefore, the goal of the present study is, first and foremost, to lay out a general developmental trajectory of the clause chaining construction in Korean children. For this goal, I examine the following aspects:

(i) Emergence and order of acquisition of conjunctive forms and their semantic functions;
(ii) Topic continuity in Korean children’s use of conjunctions;
(iii) Non-finiteness and finiteness of verbs in medial clauses;
(iv) Referential choices and their appropriate uses;
(v) Event segmentation: Types of events that children pick out and connect in clause chains;
(vi) Developmental milestones from age 3 years to adulthood in the acquisition of clause chaining construction.

The present study uses two sets of data for the investigation: longitudinal and cross-sectional. The longitudinal data are spontaneous mother–child interactions of five mother–child pairs recorded over various time periods between 1;11 and 5;1. The cross-sectional data are children’s (in five age groups) and adults’ descriptions of short animated video clips. Each video clip shows a series of object movements that are causally and temporally related. The longitudinal data are presented first, to lay out a general developmental trajectory of clause chaining in the Korean children in this sample, particularly on the development of the relation between conjunctive forms and functions (i-iii). The cross-sectional data are then presented to systematically investigate the development of clause chaining from 3 years to adulthood, particularly in relation to conjunction frequency, referential choices, length of clause chaining sentence, and segmentation of a macro event into sub-events for clause chaining constructions (iv–vi). The cross-sectional data will also be compared to the longitudinal data as appropriate, particularly to examine consistent developmental patterns.

## The Longitudinal Study

### Database

The longitudinal data come from five children, all growing up monolingually in Korean in South Korea. As shown in Table 2, the data are grouped into two sets, based on the difference in the duration of study period. More specifically, among the five children, the study period duration for Child JW was much more extensive than those of the other four, spanning 2 years starting from 1;11 (year;months). JW also produced many clause chaining utterances, so his data constitutes a full dataset, Dataset 1, in its own right. During this 2-year period (1;11–3;11), JW produces all of the 12 conjunctions studied in this paper, thus his data are the main source of developmental data for this age period. The other four children, together constituting Dataset 2, were each recorded for about 1 year starting at different ages between 2;0 and 5;1, a period that overlaps with JW’s study period and extends to the sixth year of life (i.e., 4;0–5;1). Dataset 2 supplements JW’s data in the investigation of the development of clause chaining. Consistent patterns between the two datasets, particularly with regards to the types of conjunctive form and function acquired as well as their order of acquisition will suggest that, although limited in sample size, the findings are not idiosyncratic.

**TABLE 2:** Database of clause chaining utterances in the longitudinal study.

	**Dataset 1**	**Dataset 2**			
**Child (M/F)**	**JW (M)**	**KJ (M)**	**SH (F)**	**BK (M)**	**YJ (F)**
Sibling situation	1st child with younger sister	Only child	2nd child with older sister	1st child with younger brother	2nd child with older sister
Study period years;months	1;11–3;11	2;0–2;11	3;5–4;4	3;10–4;9	4;2–5;1
Average recording time per session (min)	29.7	30.2	31.1	30.3	31.5
Total number of sessions analyzed	26	12	12	12	12
Number of clause chains (CC)	517	134	97	92	88
Total CCs per dataset	517	411			

*Each child was recorded twice a month, approximately 15 days apart between the two sessions. Of the two sessions, only the second session is included in the present study.*

The five children and their mothers participated on a voluntary basis with verbal consent. JW was recruited through the Principal Investigator’s^5^ acquaintances for a 2-year longitudinal study. The other four children were recruited on the basis of a 1-year commitment, which was already a long period for mothers to commit themselves to the study, starting at different ages, to expand the database. All five families were of middle to upper-middle class, and the parents had completed higher education, i.e., a college degree or above.

The data collection method was the same for all five children. Each child was visited at his/her home twice a month by a researcher. At each visit, spontaneous mother–child conversations were audio-recorded for 30 min (Table 2). The conversation centered around joint activities – between the mother and the child – such as book reading, pretend play, playing games (e.g., jigsaw puzzles), or conversation on topics such as the child’s everyday school experiences, past experiences, and future plans. For the purpose of the present study, only the data from the second visit of month are analyzed.

All spoken interactions were transcribed with contextual notes. Two research assistants, trained in psycholinguistics, were assigned to each child in transcribing the recorded conversations. Of the two, the principal assistant transcribed the conversations first, then the secondary assistant checked those transcriptions. When the two transcribers did not agree, the Principal Investigator resolved the conflict. Unintelligible phrases/utterances were noted as such. The transcription followed the guidelines defined in Bae (2000) for transcribing Korean children’s speech. An utterance ending was identified by rising/falling intonation and pauses of two or more seconds (Bae, 2000).

At each session, the total number of the child’s utterances – excluding one-word utterances, *ung/ney* “yes (plain/POL form)” or *ani/aniyo* “no (plain/POL form)” and immediate and full imitation of mother’s utterance - ranged between 150 and 300. In the present study, only those utterances consisting of clause chains in the transcript, as characterized in the section “Characteristics of the Clause Chaining Construction in Korean,” are examined.

Child JW produced 20 clause chains on average per session, amounting to 517 utterances as Dataset 1 for the current study (Table 2). The other four children, KJ, SH, BK, and YJ, together produced a total of 411 clause chaining utterances, much less than JW did. Although they provided less clause chaining data than JW, the two datasets converge on a coherent developmental pattern of clause chaining in terms of the types of conjunctions produced during the study periods and the order of acquisition of those conjunctions.

### Development of Conjunctive Forms and Functions

To assess a general developmental trajectory of the clause chaining construction, I first examine the types of conjunctive forms and functions that the children produced during the study periods. Table 3 shows the conjunctive forms produced in Dataset 1 (JW) and in Dataset 2 (four children), total token frequency and in percent of conjunctive forms. It also shows the onset age of each form. An onset age is identified when the child produces a given conjunctive form at least three times in the same session or in three successive sessions combined. In the latter case, the first of the successive sessions was determined as the onset age. Note that the onset ages here concern the form and not a particular function. (Development of function will be presented in the sections “Early-Acquired Conjunctive Forms and Functions” and “Later-Acquired Conjunctions”).

**TABLE 3:** Conjunctive forms, token frequency, and percentage of use.

**Conjunctive form/gloss**		**Semantic function**	**Dataset 1: JW**		**Dataset 2: four children**	
				
			**Token (%)**	**Onset age**	**Token (%)**	**Onset age**
1.	*-ko* “and”	Listing; manner; sequence; contrast	177 (34.2)	2;0	140 (34.1)	2;0
2.	*-ese* “and then”	Manner; cause; sequence	118 (22.8)	2;0	62 (15.1)	2;4
3.	*-myen* “if/when”	Condition	53 (10.3)	2;2	86 (20.9)	2;4
4.	*-nikka* “because”	Cause	28 (5.4)	2;3	18 (4.4)	2;5
5.	*-teni* “when/as”	Initiating event	26 (5.0)	3;3	0	n/a
6.	*-nuntey* “given that”	Circumstance; contrast	23 (4.4)	2;1	26 (6.3)	2;2
7.	*-ekaciko* “and then”	Sequence; cause	22 (4.3)	2;2	25 (6.1)	2;2
8.	*-le* “(in order) to”	Purpose	16 (3.1)	2;2	4 (1.0)	2;7
9.	*-taka* “while doing”	Interrupted event	12 (2.3)	2;7	6 (1.5)	2;9
10.	*-ulttay* “when”	Temporal frame	6 (1.2)	3;10	12 (2.9)	3;11
11.	*-myense* “while”	Simultaneity	6 (1.2)	3;4	8 (1.9)	2;10
12.	*-eto* “though”	Adversity	6 (1.2)	2;11	5 (1.2)	2;11
	Other^1^		24 (4.6)		19 (4.6)	
	Total		517 (100)		411 (100)	

*^1^Other conjunctions include *-lyeko* “in order to,” -*key* “so that,” *-ntaumey* “after.” *-yaci* “if/when,” *-eya* “only when,” *-sunkaney* “at the moment of,” which are produced between two and four times in a dataset. In addition, nine other conjunctions are produced, but each occurs once in a dataset.*

Comparing between the two datasets, both the repertoire of the conjunctive forms and their frequency distributions are similar and converge on a coherent developmental pattern in terms of the acquisition order. Specifically, the first three most frequent conjunctive forms, *-ko* “and,” *-ese* “and then, because” and *-myen* “if/when,” are the same in both datasets. These three forms together constitute about 67% (Dataset 1) to 70% (Dataset 2) of the children’s production of conjunctions. The next four forms on the list, *-nikka* “because,” *-teni*, “when,” *-nuntey* “given that” and *-ekaciko* “and then,” are produced much less frequently (than the first three forms), each ranging between 4 and 6% in each dataset. One exception is the conjunction *-teni* “when,” which denotes an initiating event to the main event. Note that the initiating event with *-teni* “when” triggers the main event but does not directly cause it. (See the example of *-teni* in Table 1 and also the section “From 2;7 to 3;11: Acquisition of complex temporal concepts”). JW produces it 26 times (5.0%), but no children in Dataset 2 use it during the recording sessions.^6^ The third set of conjunctions in Table 3 are *-le* “(in order) to,” *-taka* “while doing,” *-ulttay* “when,” *-myense* “while,” and *-eto* “though.” These forms are produced with frequencies of 3% or less in each dataset. Regarding this last set of conjunctions, there are some differences between the two datasets. JW (Dataset 1) uses *-taka* “while doing” and *-le* “(in order) to” relatively more often than the other four children. On the other hand, the temporal frame marker, *-ulttay* “when,” is more frequent in Dataset 2 than in JW’s speech.

Overall, the children produce the multi-functional conjunctions, *-ko* and *-ese*, most frequently. They also produce the conjunction *-myen* “if/when” frequently denoting a condition to the main event. The degree of frequency in children’s production of conjunctions probably relates, at least in part, to the frequency rates in caregivers’ speech. While it is beyond the scope of this paper to examine the mothers’ frequency distribution of the conjunctions, the frequency data of conjunctions in the adult narrative data reported in Kim (1992) can be compared to the children’s data. The six most frequent conjunctions in Kim’s data are, *-ko* “and then,” *-ese* “and then, because,” *-nuntey* “but,” *-nikka* “because,” *-taka* “while doing,” and *-myense* “while.” Four of the six forms, *-ko* “and then,” *-ese* “and then, because,” *-nuntey* “but,” and *-nikka* “because,” are also ranked relatively high in the children’s data for the current study (Table 3), supporting the input hypothesis to some degree. Further studies are necessary to examine the extent of the input frequency factor in children’s development of conjunctions.

A coherent developmental pattern also emerges in terms of the *onset ages* of the forms, which are reported in Table 3. The 12 conjunctions can be divided into two groups according to their onset ages. In the first group, six of the first seven on the list, namely, -*ko* “and then,” *-ese* “and then, because,” *-myen* “if/when,” *-nikka* “because,” -*nuntey* “but,” and *-ekaciko* “and then,” appear in the children’s production earlier than 2;6 in *both* datasets. The conjunction *-le* “(in order) to,” appear at 2;2 in JW’s speech, but only at 2;7 in Dataset 2. Not surprisingly, these conjunctions also rank high in frequency. The second group includes the remaining five conjunctions: *-teni* “when/as,” *-taka* “while doing,” *-ulttay* “when,” *-myense* “while,” and *-eto* “though,” which appear after 2;7. In order to explain the particular order of acquisition of the forms, we need to look at their semantic functions and assess which types of meanings children convey earlier than others. Thus, in the following, I examine the development of the 12 conjunctions in terms of their semantic functions, starting with the earlier-acquired forms.

#### Early-Acquired Conjunctive Forms and Functions

Among the seven early-acquired forms, *-ko* “and then,” *-ese* “and then, because,” *-nuntey* “given that, but,” and *-ekaciko* “and then, but” serve several functions (Table 1). I will first present how the functions develop in these multi-functional forms before going on to the other early-acquired forms. For each multi-functional form, I analyzed the semantic function expressed by the form in relation to the next clause, which is typically the final and main clause. Then, I determined the onset age of each function for the given form using the same criteria as those for the forms, i.e., the first session (or the first of the three successive sessions combined) in which the particular function of the form was expressed at least three times. Table 4 presents the results of the analysis along with the token frequency of each function for the form.

**TABLE 4:** Conjunctive forms and functions, onset age and token frequency of each function.

			**Onset age (token frequency)**	
**Conjunctive form/gloss**		**Semantic function**	**Dataset 1**	**Dataset 2**
*-ko*	‘and’	Listing	1;11 (61/177)	2;0 (29/140)
	‘by ∼ing’	Manner/means	2;0 (62/177)	2;1 (55/140)
	‘and then’	Sequence	2;4 (36/177)	2;4 (43/140)
	‘but’	Contrast/juxtaposition	2;6 (12/177)	2;8 (7/140)
*-ese*	‘because’	Cause	2;0 (72/118)	2;4 (25/62)
	‘by ∼ing’	Manner	2;0 (17/118)	2;4 (20/62)
	‘and then’	Sequence	2;1 (29/118)	2;4 (17/62)
*-ekaciko*	‘and then’	Sequence	2;2^1^ (10/22)	2;2^1^ (17/25)
	‘because’	Cause	3;2 (12/22)	4;1 (8/25)
*-nuntey*	‘given that’	Circumstance	2;1 (15/23)	2;2 (5/26)
	‘but’	Contrast	2;4 (7/23)	2;2 (21/26)
*-nikka*	‘because’	Reason	2;1 (28)	2;5 (18)
*-myen*	‘if/when’	Condition	2;2 (53)	2;4 (86)
*-le*	‘(in order) to’	Purpose	2;2 (16)	2;7 (4)
*-taka*	‘while doing’	Interrupted event	2;7 (12)	2;9 (6)
*-eto*	‘though’	Adversity	2;11 (6)	2;11 (5)
*-ulttay*	‘when’	Temporal frame	3;1 (6)	3;11 (12)
*-teni*	‘when/as’	Initiating event	3;3 (26)	None
*-myense*	‘while’	Simultaneity	3;4 (6)	2;10 (8)

*^1^See the text for details of the developmental pattern of *-ekaciko* with a temporal function.*

##### *-ko* expressing list,^7^ manner, sequence, and contrast

The conjunction *-ko* fulfills several functions: listing individual events/states (9), specifying the manner of an activity, and providing sequential, or contrastive information (10–13) in relation to the main clause. The frequency distribution of these functions in Table 4 shows that the listing, the manner, and the sequential functions are prominent in the children’s use *-ko*. In each dataset the four functions appear at different times but with same order of appearance. The conjunction *-ko* “and” is first used to simply list events/actions/states as in (9), and then a month later to provide manner information (10–11). In the manner function, *-ko* specifies the means by which the activity in the main event is performed, in particular the tool that one uses or carries to perform a task (10) or the type of vehicle one uses for a spatial motion (11). Such tool and vehicle specifications occupy 80% of all *-ko* uses with a manner function.


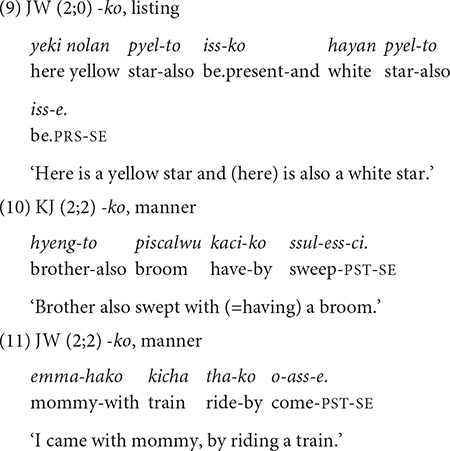


The sequential and contrastive functions of *-ko* “and then, but” appear a few months later (12–13). For the sequential function of *-ko* “and then,” the non-main event [e.g., watching TV in (12)] happens first, before the main event takes place. However, it is important to note that a non-main event with the conjunction *-ko* is not a pre-requisite/pre-conditional activity for the main event to happen (Sohn, 2009). Furthermore, the non-main event can occur in space and time different from those of the main event. The relationship between the non-main and the main event is somewhat “loose,” to use Longacre’s (1985) term (p. 177). As we will see, this relational aspect of *-ko* contrasts with the sequential function of *-ese* “and then.”


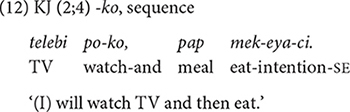


The contrastive function of *-ko* “but (instead)” is the last one to appear. It is also the least-frequently expressed function of *-ko*. In this function, the speaker juxtaposes two alternative activities/events with *-ko* on the medial clause, favoring one over the other (13). All of the children’s *-ko* utterances with a contrastive function show such juxtaposition with a NEG particle in the medial clause. For example, in (13), JW juxtaposes the activities of watching TV and reading books, and suggests not watching TV but reading instead. The late appearance of this function may be due to its underlying concept being relatively more abstract than the other functions of *-ko*. More specifically, while the manner and sequential aspects of events are perceptually iconic, a contrastive aspect – with negation – between activities is more conceptual.


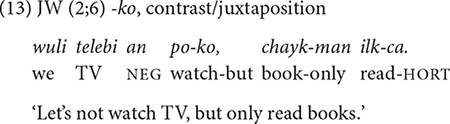


##### *-ese* expressing manner, sequence, and cause

The conjunction *-ese* is another major form in Korean. It specifies manner (14), sequential (15), and causal (16) aspects of the event in the main clause. In contrast to the developmental pattern of *-ko*, the children in both datasets use all three functions of *-ese* around the same time (Table 4). JW does so within a month, between 2;0 and 2;1, and KJ, the youngest child in Dataset 2, in the same session at 2;4.


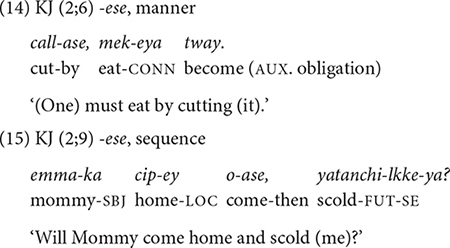


Similar to *-ko*, the conjunction *-ese* expresses manner as well as temporal sequence. However, a closer look at the semantic relations they express with respect to the main event suggests that there is a division of labor between them. In fact, the two conjunctions are often not interchangeable. Regarding manner, we have seen that the conjunction *-ko* specifies the tool or the vehicle used (10–11). In sharp contrast, none of the *-ese* clauses express such a function. Instead, with *-ese*, the medial clause specifies the manner of motion by which one achieves the main event, as in (14). It also specifies one’s posture (e.g., lying, sitting) or the path (e.g., going around) to accomplish an action. The two conjunctions, *-ko* and *-ese*, also differ regarding their temporal sequence functions. The relationship between the non-main and main events is much tighter with the *-ese* conjunction than with *-ko* (see the section “-*ko* expressing list, manner, sequence, and contrast”). With *-ese*, the event in the non-main clause is a pre-condition for the main event, in that the main event takes place in the state produced by the event of the *-ese* clause (Kim, 1992; Sohn, 2009). For example, in (15), KJ asks his mother whether she’ll scold him when she comes home, which means that the mother has to come home first for the scolding to take place.

The conjunction *-ese* also has a causal meaning as in the following examples. The causal meaning is prominent in JW’s use of the form. In (16), JW’s mother cries because JW is absent and in (17), BK wipes because he spilled something.


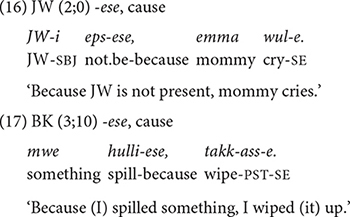


Notice that in all three functions of *-ese* (14–17), the non-main and main clauses have close temporal proximity. The main event follows almost right after the non-main event takes place. It is possible that this common feature of the three functions facilitates children’s acquisition of all three functions at around the same time. On the other hand, as mentioned earlier, input frequency is another possible factor for the early acquisition of these functions. As we will discover later (section “Type and Token Frequency of Conjunctions by Age Group”), *-ese* is by far the most frequent conjunction that adults use to express manner, cause, and temporal sequence.

##### *-ekaciko* expressing sequence and cause

The form *-ekaciko* “and then” is a morphologically transparent form. It consists of the verb *kaci*-, which means “to have/possess.” A literal translation of V*-ekaciko* would be “having done X.” As a conjunction, it expresses temporal or causal sequence, quite similar to the functions of *-ese* “because, and then.” In fact, the two forms, *-ekaciko* and -*ese* are often interchangeable. Thus, (18a) and (18b) below have same meaning and both are acceptable. But the developmental trajectory of *ekaciko* is different from that of *-ese* (Table 4). In both datasets, the children produce *-ekaciko* “and then” several times between 2;2 and 2;3 to express temporal sequence. However, after 2;3, there is a long period of absence of the form (with a temporal meaning) in their speech. Then, the sequential function of *-ekaciko* “and then” reappears sporadically at around 2;8, and more consistently at 3;10 in dataset 1 and at 3;7 in dataset 2 (19). Also, the onset of *-ekaciko* “because” as a causal function is much later than *-ese* “because.” The rather inconsistent and late uses of *-ekaciko* may have to do with the fact that the same functions can be expressed by *-ese*, which the children have already acquired. They may also have to do with input frequency in mothers” speech. Later in the section “Type and Token Frequency of Conjunctions by Age Group,” the cross-sectional data will reveal that adults do not use *-ekaciko* in describing a series of motion events connected by cause and temporal sequence, whereas children do.


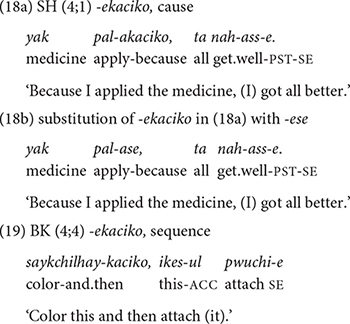


##### *-nuntey* expressing circumstance and contrast

The conjunctive form *-nuntey* “given that, but” has circumstantial and contrastive meanings. Circumstantial meaning refers to providing background or contextual information of the main event, thus are less tied to immediate action sequences. The children produce both functions before 2;6, but the frequency of the two functions differs in the two datasets. From 2;0, JW uses it for a circumstantial meaning (15 times out of 23, Table 4) as he describes events from a story book (20). It is worth noting that JW uses *-nuntey* in this way almost exclusively (13 out of 15 times) when narrating stories from story books (e.g., Pinocchio, Winnie-the-Pooh), the kind of activity he often engaged in with his mother. JW seems to have acquired the circumstantial function of *-nuntey* through book reading.


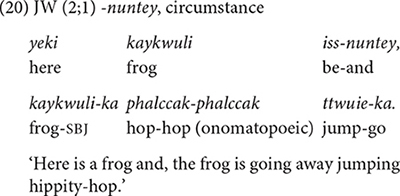


JW starts expressing the contrastive function with *-nuntey* a few months later (2;4) and he expresses the function only occasionally (7 times out of 23). In contrast, the four children in Dataset 2 show the opposite pattern. KJ, the youngest child in the dataset, uses *-nuntey* for a contrastive function from 2;2. He produces a total of seven utterances with *-nuntey* during the study period, and most of them — five out of seven — are contrastive (21). Similarly, SH (3;5–4;3) produces *-nuntey* six times, five of which are contrastive and only one circumstantial. In BK’s speech (3;10–4;9), all six *-nuntey* utterances are contrastive (22). The oldest child, YJ (4;2–5;1), produces seven *-nuntey* utterances with five of them in contrastive meaning. Note that the contrastive function of *-nuntey* is different from that of *-ko*. In the case of *-ko*, the function is to juxtapose two alternatives (section “-*ko* expressing list, manner, sequence, and contrast”), but *-nuntey* signals that the main event is contrary to a customary assumption.


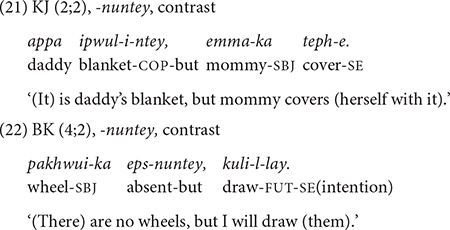


In both datasets the early utterances with *-nuntey* are sometimes not adult-like. In some cases, it was used for a listing or sequential function for which the form *-ko* “and then” would have been more appropriate. Example (23) illustrates such a case. Inappropriate uses of *-nuntey* at early stages suggest that the children do not fully acquire the relevant concept of circumstance until later.^8^


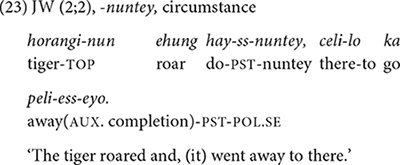


##### *-nikka* expressing a logical reason

Although the semantic function of *-nikka* can generally be labeled as “cause” (Sohn, 2009), it is quite different from other conjunctions expressing “cause” (Table 3). For example, unlike *-ese*, which typically expresses a cause whose effect is spatially and temporally related to the main clause (15), *-nikka* expresses some logical reason that justifies an event. This is demonstrated in the children’s use of the form in (24–26). JW starts using *-nikka*, at 2;1, KJ in Dataset 2 starts a little later at 2;5.


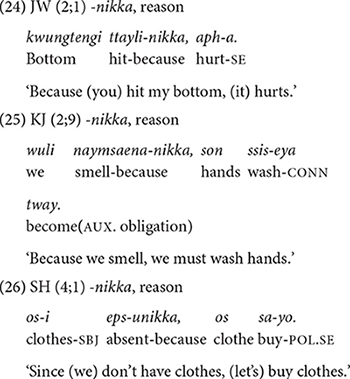


Although the concept of “reason” is abstract, interestingly, there are no inappropriate uses of -*nikka*. The concept of reason, which relates to that of cause, may be cognitively more accessible than the concept of circumstance.

##### *-myen* expressing condition

The children produce the conjunction *-myen* “if/when” from early on, 2;2 in JW’s speech and 2;4 in Dataset 2 (Table 3). They produce it frequently and appropriately to express a condition for the main event, as in (27) and (28).


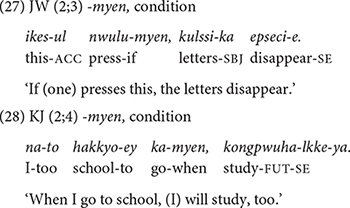


The early appearance of conditional expressions in Korean children has been well-documented in previous research. Akatsuka and Clancy (1992) report that Korean (and Japanese) children express conditionals as early as 2;0, and contrast their findings with a relatively later acquisition (2;6–3;0) of the conditional “if” in English (Bowerman, 1986; Reilly, 1986) and in Italian and German (Clancy et al., 1976). Akatsuka and Clancy (1992) explain that while the concept of condition is abstract, it may be more accessible to Korean children because the conditional conjunction in Korean, *-myen*, also occurs with a deontic modal verb, *(an-)tway* “(not-)good” to refer to social norms as in (29), and mothers often appeal to children using this construction.


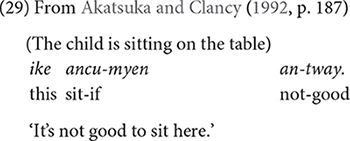


##### *-le* expressing purpose

At 2;2, JW also produces *-le* “(in order) to” 16 times to express a purpose of an action, but in Dataset 2, KJ uses it three times at 2;7 and SH uses it only once at 3;6. In all cases, the conjunction is followed by a deictic motion verb, *-ka* “go” and *-o* “come” (Table 1), stating the purpose of going somewhere. This restriction also applies in the adult grammar (Sohn, 2009). The children begin to produce the morphologically more elaborate and syntactically less restrictive conjunctive form *-lyeko* “in order to” much later, after 3;6, to express purpose.

In summary, by 2;6 the children in this sample produce several major types of conjunctive forms that express cause, reason, manner, sequence, circumstance, contrast, and condition. Impressively, from the beginning young children use the forms appropriately according to the specific form–function relationship in the adult grammar.

#### Later-Acquired Conjunctions

##### From 2;7 to 3;11: acquisition of complex temporal concepts

From 2;7, children produce five new conjunctions, *-taka* “while doing,” *-teni* “when/as,” *-myense* “while, *-ulttay* “when,” and *-eto* “though.” Except for *-eto* “though,” all conjunctions relate to temporal concepts and are qualitatively different from the earlier-acquired ones. These later-acquired conjunctions express some type of deviation from smooth successive actions/events. More specifically, the conjunction *-taka* “while doing” expresses a change of course in action/movement (30). As for *-teni* “when,” it expresses an initiating event that is only loosely connected to the ensuing event. That is, the event that follows could be unrelated or unexpected (31) from the *-teni* event. The conjunction -*myense* “while” presents an event that overlaps in time with the main event (32), and *-ulttay* “when” sets up a general temporal frame for an event that is not tied to a specific event (33). I would argue that these notions are cognitively more demanding than the earlier-acquired temporal concepts, in that they connect disjunctive/interrupted actions (*-taka, -teni*), express a general temporal relation of two events that are not tied to the here-and-now (*-ulttay*), or convey the temporal overlapping of two events (*-myense*) that are woven into a single macro event (Slobin, 1995).


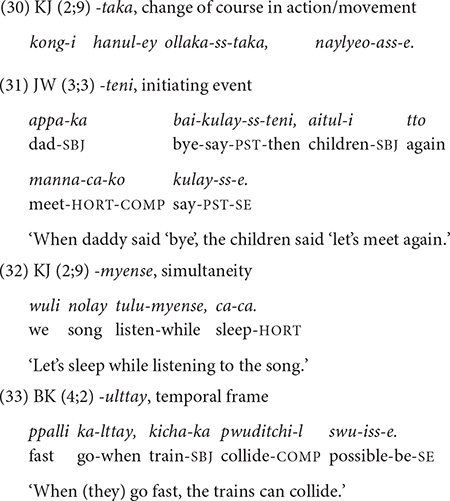


Toward 3;0, the children start using the conjunction *-eto* “although” which denotes concessive. In (34), JW expresses his brave and unexpected reaction to falling down. The concept of concessive expressed in *-eto* (i.e., despite a difficult situation, …) may be likened to that of change/disjunction mentioned above for the later-acquired temporal conjunctions, in that the reaction to an adverse situation does not follow the usual expectation.


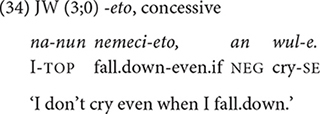


##### From 4;0 till 5;1

Here I briefly comment on the clause chaining data of the two oldest children in Dataset 2, BK and YJ, whose speech was recorded between 3;10 and 5;1. Essentially, there are no new developmental aspects that are qualitatively different from the younger children. The two children continue to produce the 12 conjunctions at similar rates as the younger children, except for using the general temporal frame marker, *-(u)lttay* “when” more productively (35). One new conjunction that appears in their repertoire is *-key* “so that,” expressing permission, causation, or purpose (36), which they produce only three times in total. We will discover that the absence of significant development beyond 4 years converges with the elicitation data.


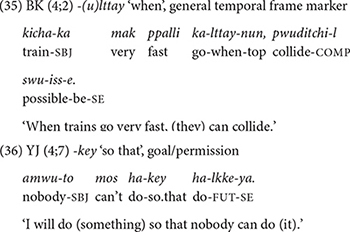


### Topic Continuity Feature of Conjunctions

As mentioned in the section “Introduction,” many of the clause chaining conjunctions in Korean have clear preferences in regards to whether or not the following clause has the same subject (SS). For example, the simultaneity conjunction *-myense* “while” has a strong tendency to maintain the SS in the main clause as in (32) above, whereas the circumstantial conjunction *-nuntey* “given that” freely allows a different subject (DS) in the main clause as in (37). In Kim’s (1992) adult data the temporal conjunctions *-myense* “while,” *-taka* “while doing,” and *-ese* “and then” had a 90% rate of SS, whereas *-nuntey* “but” and *-nikka* “because” had only a 18.2% rate of SS.


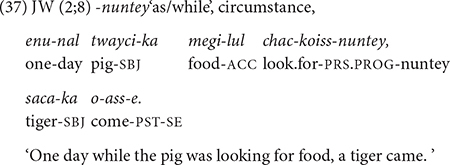


An analysis of topic continuity was carried out to determine whether the children in the present study already use conjunctions with a clear preference for DS or SS, similar to the way adults use them. The result shows that children do so in large part. I should note here that in the children’s speech, the subject of a clause was often omitted (zero anaphora) but identifiable, as the children talked about themselves, their mothers, or the things that they were playing with. (I will take up the issue of referential choices with cross-sectional data in the section “Referential Ambiguity of Subject Argument”). Table 5 shows the conjunctive functions, the forms used, and their SS and DS frequency distributions in the children’s speech. First, as in the adult narratives reported in Kim (1992), all the conjunctions expressing temporal sequence, simultaneity, and manner, e.g., *-ko* “and then,” -*myense* “while,” clearly prefer SS. In contrast, conjunctions marking a circumstance, contrast, condition, and reasoning prefer DS. The listing conjunction *-ko* “and” also allows the possibility of switching to a DS in the main clause. The events serving these functions are often external to the main event. By “external,” I mean that events explaining circumstance, contrast, etc., are not integrated parts of what happens in the main event. Note that the preference for SS or DS, i.e., the topic continuity feature, depends on the conjunctive function, not the form. For example, the sequential function of *-ko* is predominantly associated with SS, but the listing function is associated more with DS than SS.

**TABLE 5:** Topic continuity of conjunctions: Frequency of different (DS) and same subject (SS).

**Semantic function/DS/SS preference**	**Conjunctive form**	**DS vs. SS (token frequency)**	
		**Dataset 1**	**Dataset 2**

**SS preferred**			
Sequence/manner	*-ko*	DS (13) < **SS^1^** (97)	DS (9) < **SS** (89)
Sequence/manner	*-ese*	DS (3) < **SS** (43)	DS (4) < **SS** (33)
Sequence	*-ekaciko*	DS (2) < **SS** (8)	DS (0) < **SS** (17)
Simultaneity	*-myense*	DS (0) < **SS** (5)	DS (0) < **SS** (8)
Interrupted event	*-taka*	DS (0) < **SS** (12)	DS (0) < **SS** (5)
**DS preferred**			
Listing	*-ko*	**DS** (34) > SS (27)	**DS** (19) > SS (10)
Circumstance/contrast	*-nuntey*	**DS** (17) > SS (5)	**DS** (17) > SS (9)
Reason	*-nikka*	**DS** (22) > SS (5)	**DS** (13) > SS (5)
Condition	*-myen*	**DS** (33) > SS (17)	**DS** (61) > SS (24)
Initiating event	*-teni*	**DS** (16) > SS (4)	No data
**Indeterminate**			
Temporal frame	*-ulttay*	DS (3) = SS (1)	DS (2) < **SS** (10)
Cause	*-ese*	**DS** (43) > SS (29)	DS (11) = SS (14)
Cause	*-ekaciko*	**DS** (9) > SS (3)	DS (2) = SS (3)

*^1^The boldface refers to the type (DS or SS) that is clearly preferred in the dataset.*

The third category of functions in Table 5, namely, *-ulttay* “when,” *-ese* “and then, because,” and *-ekaciko* “and then,” do not show a clear preference. The two sets of data differ somewhat in terms of their preference for SS or DS, and in some cases (i.e., *-ulttay, -ekaciko*), the amount of data is too small to make generalization. We will examine the topic continuity feature further in the cross-sectional study.

### Non-finiteness Versus Finiteness of Medial Verbs

In the children’s speech, non-finiteness of the verbs in medial clauses is well observed. Of all the verbs in medial clauses, only 8.5% (44/517) for JW and 3.9% (16/411) in Dataset 2 are finite. The finite verbs typically carry the past tense inflection, *-ess* (Kim, 2015). In the data, finite verbs in medial clauses are restricted to the following conjunctions: *-teni* expressing an initiating event (in all 20 cases), *-nuntey* expressing a circumstance/contrast (24 cases out of 50), *-taka* expressing an interrupted event (8/17), *-nikka* expressing a reason (8/45), and *-ko* (2/90). In JW’s speech all the verbs with *-teni* “when/as” is finite, as in (31), repeated here. Also, half of the verbs with *-nuntey* “but” are finite (38).


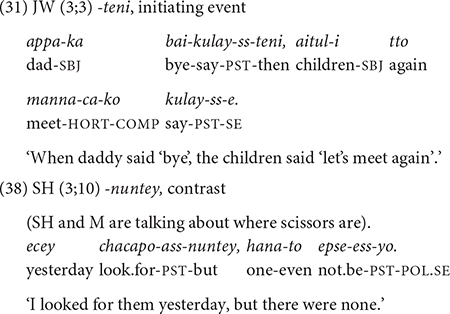


What these events with finite verbs have in common is that the medial, non-main clause is temporally not always continuous with the event in the main clause. In this regard, interestingly, the verbs with conjunction *-ko* in a listing function – which can connect individual events/states – are finite only in two cases out of 90 occurrences. A close look at the data show that with *-ko* the children typically list object names or various states of affairs that they observe at the time of speech, often with adjectival predicates, as in (9), repeated here. This may explain the scarcity of finiteness (2/90) in the verb with conjunction *-ko* in the children’s speech.


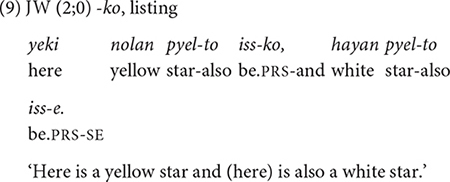


### Summary of the Longitudinal Study

The Korean children in this sample combine clauses from at least 2 years of age. Within 6 months, they acquire eight prominent types of conjunctive forms and use those linguistic devices appropriately to specify the cause, manner, sequence, contrast, and condition of the main clause. Several of the conjunctions are multi-functional and some functions can be served by more than one form, and yet, from this early stage, children are strikingly good at using specific forms for specific functions with few errors. At the beginning (2;0–2;6), many of the functions that children express are based on concrete and perceptual events having cause–effect and sequential relations, particularly with *-ko* “and” and *-ese* “and then, because.” But during the next year or so, they produce temporal conjunctions that are cognitively more demanding. They begin to express action sequences that have interruptions or a change of direction in the course of an action. They also express the concepts of the simultaneity of events and of having a general temporal frame for an event. By 5;0, children have a repertoire of as many as 25 conjunctions. Children’s proportions of topic continuity feature for each conjunction are similar to those of adults. As in the adult grammar, the verbs in medial clauses are mostly non-finite, except with those conjunctions which convey an event that is temporally distinct from the main event.

I turn now to the second part of the paper and report further developmental aspects of clause chaining based on a cross-sectional elicitation study. I also compare the elicitation data to the longitudinal data.

## The Cross-Sectional Elicitation Study

In this elicitation study, children of various age groups (from 3 to 10 years) and adults described short video clips of animated motion events that involve chains of events where one object affects another object in a particular way. As it is a controlled study with the same sets of stimuli, we can systematically investigate the following aspects in the development of clause chaining in Korean: type and token frequencies for temporal and causal conjunctions, the topic continuity feature of conjunctions, and the development of referential choices. We can also examine children’s ability to segment a macro event into sub-events, which can be connected in a clause chain.

### Materials and Methods^9^

#### Participants

A total of 80 monolingual Korean learners/speakers participated in the study as part of a larger study that was designed to elicit motion event expressions involving different manners (e.g., sliding, running) and paths (e.g., up, into) of motion. Of the 80 participants, data from eight child participants (three 3-year-olds, four 4-year-olds, and one 6-year-old) were excluded from analysis for one of the following reasons: (i) the child stopped describing after a few stimuli items due to apparent boredom (three 4-year-olds), (ii) the child did not want to talk to the experimenter from the beginning (two 3-year-olds and one 4-year-old), (iii) the child told stories from his/her own imagination that had nothing to do with the stimuli (one 6-year-old), and (iv) the child told the same sentence over and over again for all stimuli, e.g., “it went like this and like this” (one 3-year-old). As a result, data from 72 participants, 12 children in each of five age groups – 3, 4, 6, 8, and 10 years – and 12 adult speakers, were included in the analysis. The adult participants were university students between 22 and 30 years of age. All data were collected in Seoul, South Korea. The adult participants and parents/guardians of the child participants signed written consent forms. The adult participants were given monetary compensation and the children were given stickers and books for their participation.

#### Materials and Design

For the present study, a total of seven video clips of animated motion events were created. Each video clip was 4 s long and consisted of three to five sub-events, which were temporally and causally related as in the following example: (see Supplementary Appendix A for a full description of all seven events).


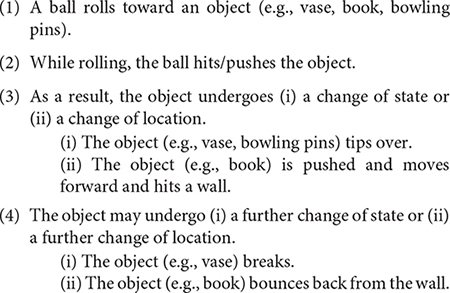


**FIGURE 1:**
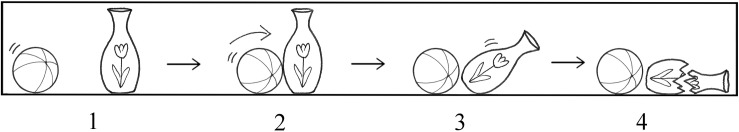
Example of a series of sub-events within a video event in the elicitation experiment. Each video event are 4 s long and the series of sub-events occur without any pauses. The figure illustrates the video event involving a ball and a vase: **(1)** The ball rolls, **(2)** hits the vase, **(3)** the vase tips over, and **(4)** the vase breaks. As shown, the sequence from one sub-event to the next involves causal and temporal relations. Each sub-event involves movement, contact with another object, or change of state/location.

Figure 1 illustrates the video event involving a ball and a vase. As shown, the connection from one sub-event to the next involves causal and temporal sequence. Each sub-event involves a movement or a change of state: for example, the ball rolls and the vase breaks. In the experiment, the seven video clips were interspersed with 32 other stimuli videos of animated motion events (e.g., A boy walks up the hill carrying a bag), which are not included in the present study.

#### Procedure

The participant was presented with one video clip at a time on a computer screen. After each clip, he/she was asked to tell what happened in the video. Adults and children in age groups of 6, 8, and 10 years were asked to describe the video events to an imagined friend who hadn’t seen them. For 3- and 4-year-olds, a doll named Ppororo (a popular animated figure in a Korean TV series for children) was introduced. Ppororo had big dark sunglasses on. (In the TV show, Ppororo always wears big glasses). The child was told that Ppororo could not see the screen and would like to know what happened.

#### Coding and Analysis

All descriptions were audio-taped and later transcribed by a Korean native speaker. The following are some examples.


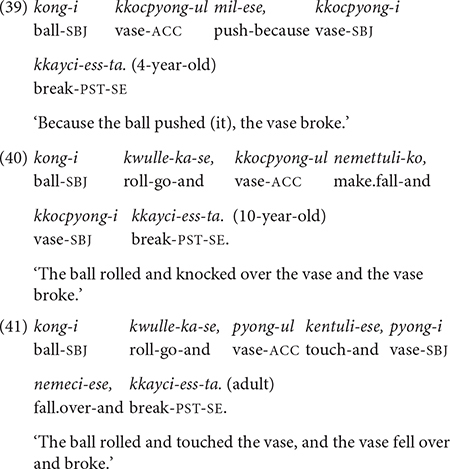


For each description, the number of clauses and the sub-events the clauses described were identified. For multi-clausal descriptions, the conjunction forms and their semantic functions were coded as well as their topic continuity feature. For each clause, the morphological nature of the subject argument – full NP, zero anaphora, or pronoun – was coded. Cases of zero anaphora were examined to determine whether they were used appropriately (i.e., clear vs. ambiguous reference) and whether the intended referent had been mentioned in a preceding clause. All verbs in medial clauses were coded with respect to their finiteness.

## Results

### Type and Token Frequency of Conjunctions by Age Group

Table 6 shows the conjunction types and their token frequencies by age group. Regarding types, a total of 15 conjunction types are produced to link sub-events, ranging from 7 to 12 types per age group. Of the 15 types, the first eight make up 96.2% of all conjunctions. The conjunction -*ese* “and then, as” is by far the most frequent (279 out of 556 total conjunction tokens, 50.2%), not only for all age groups combined but also within each age group as well. The remaining seven types are used only occasionally by various age groups. Adults produce 12 different types, adding two new conjunctives (*-nhwuey* “after,” *-npalamey* “as result of”) to the children’s repertoire, but those “new” types are each used only once. On the other hand, no adults use *-ekaciko* “and then” (expressing cause and sequence), while some children aged between 4 and 10 years do. As noted in the section “*-ekaciko* expressing sequence and cause,” during an early period, the children in the longitudinal data use the conjunction *-ekaciko* “and, because” (literally, *-e* + *kaciko* “CONN + having done”) only sporadically, and start using it again in their fourth year. The elicitation data suggest that the form becomes a less important one over time. Instead, adults express cause and temporal sequence with the morphologically opaque – thus more abstract – form, *-ese*, “and then, because” or its phonologically reduced form *-e(se)*.

**TABLE 6:** Conjunction types and token frequency by age group: cross-sectional study.

**Conjunctive form/gloss**		**3 years**	**4 years**	**6 years**	**8 years**	**10 years**	**Adults**	**Total**	**% usage**
1.	*-ese* ‘and then, as’	20	26	37	40	70	86	279	50.20
2.	*-ekaciko* ‘and then’	1	16	12	24	14	0	67	12.10
3.	*-taka* ‘while doing’	4	7	7	7	9	11	45	8.10
4.	*-ko* ‘and then’	1	3	11	5	6	14	40	7.20
5.	*-nuntey* ‘but’	1	7	11	6	5	6	36	6.50
6.	*-myense* ‘while’	0	5	0	5	8	10	28	5.00
7.	*-e(se)* ‘and then, as’	1	0	0	1	3	19	24	4.30
8.	*-teni* ‘when’	2	8	0	3	0	3	16	2.90
	Subtotal							535	96.20

9.	*-ca* ‘as soon as’	0	0	0	0	3	5	8	
10.	*-nikka* ‘because’	1	0	1	1	0	1	4	
11.	*-ntaumey* ‘after’	0	0	0	1	1	1	3	
12.	*-myen* ‘when’	1	0	1	0	0	0	2	
13.	*-ko(na)se* ‘after’	0	0	2	0	0	0	2	
14.	*-nhwuey* ‘after’	0	0	0	0	0	1	1	
15.	*-npalamey* ‘when, as’	0	0	0	0	0	1	1	
	Subtotal							21	3.80

	Grand total	32	72	82	93	119	158	556	100
Number of conjunction types		9	7	8	10	10	12		

As Table 6 shows, 3-year-olds already use all the major types of clause chaining conjunctions to express cause and temporal sequence, except *-myense* “while,” which denotes simultaneity. The later appearance of *-myense* is consistent with the longitudinal data of the present study (see the section “Later-Acquired Conjunctions”). In the cross-sectional study, *-myense* appears in the 4-year-old group. Children in the longitudinal study begin using *-myense* at around 3 years of age (2;10–3;4), but they may need more time to have sufficient control over it to use it in a controlled study. While the repertoires of major conjunctive types are similar across the age groups, their token frequencies are noticeably different: Three-year-olds produce 32 conjunctions in total (all children combined) but 4-year-olds produce twice as many conjunctions. Ten-year-olds and adults each produce 119 and 158 tokens, respectively.

**FIGURE 2:**
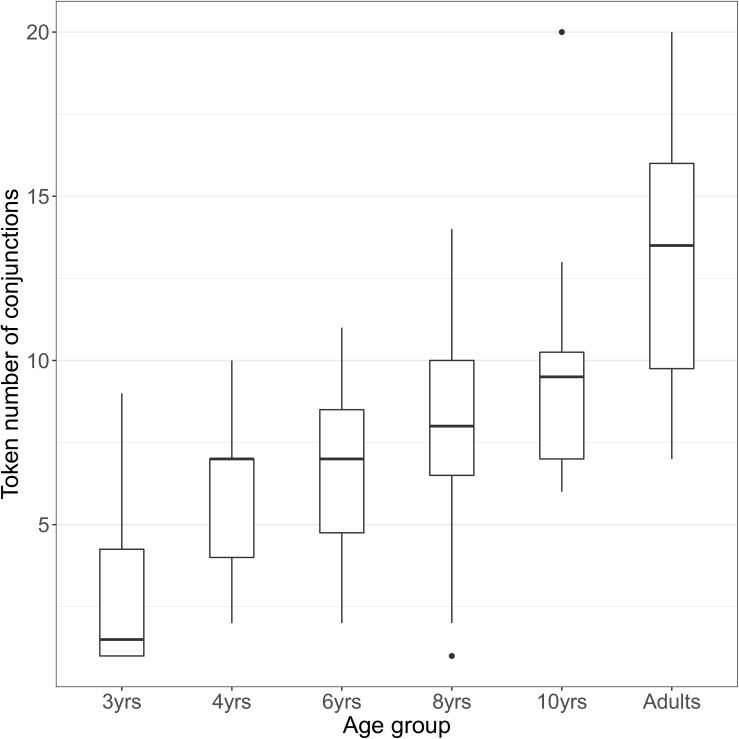
Distribution of token frequencies of conjunctions (for the seven video events combined) per participant in each age group (*N* = 12). The median frequency in each age group is marked by a thick horizontal lines that typically falls within the box. The bottom and top of the box represent the first and the third quartile (Q1 and Q3), respectively, and the two ends of the whiskers (vertical lines) represent minimum and maximum number of tokens produced by a participant in that age group. The dots represent outliers. Token frequencies grew as a function of age. See text for statistical results comparing across age groups.

Figure 2 displays box plots showing the distribution of token frequencies of conjunctions (for the seven video events combined) produced per participant in each age group. As can be seen, most 3-year-olds produce four or less conjunctions while 4-year-olds produce more than four tokens. Then, we notice little change in the distribution from 4 to 8 years except that 8-year-olds show more individual variability than the younger children. Ten-year-olds are qualitatively different from 8-year-olds: They produce more conjunctions with less individual variation. Adults produce substantially more conjunctions but also show individual variation (between 7 and 20 conjunctions). A linear model analysis using R statistics (Version 3.5.1, R Core Team, 2018) shows that the difference in the amount of conjunctions between 3 and 4 years is significant (β = 3.091, SE = 1.375, *t* = 2.248, *p* = 0.028). The next two age groups, 6 and 8 years produce about the same amount of conjunctions as 4-year-olds (Figure 2). Then, from 8 years to adults, there is a steady increase in conjunctions, i.e., medial clauses (93, 119, and 158, respectively, Table 6). While the difference between 8- and 10-year-olds is not significant (β = 2.167, SE = 1.571, *t* = 1.416, *p* = 0.177), the difference between 10-year-olds and adults shows a strong trend of increase (β = 3.250, SE = 1.592, *t* = 2.041, *p* = 0.053). Also to note, 10-year-olds produce significantly more conjunctions than 4-year-olds (β = 3.916, SE = 1.391, *t* = 2.816, *p* = 0.006) and 6-year-olds (β = 3.083, SE = 1.472, *t* = 2.094, *p* = 0.042). Overall, the data show significant or substantial increases of conjunctions from 3 to 4 years, then at 10 years, and again for adults. The increases in number of conjunction tokens means that correspondingly, older children and adults produce many more medial clauses, hence express more sub-events than younger children. Given the finding that the major conjunction types are already present in 3-year-olds” descriptions (Table 6), the data suggest that their challenge is to put several events together into one utterance.

### Topic Continuity Feature of Conjunctions: Cross-Sectional Study

In the section “Topic Continuity Feature of Conjunctions,” we saw that the temporal conjunctions denoting sequence and simultaneity prefer SS whereas the listing and contrastive conjunctions prefer DS. But for the causal conjunctions, *-ese* “and then, because” and *-ekaciko* “and then,” their topic continuity preference could not be determined (Table 5). To compare with the longitudinal data, Table 7 shows the DS and SS frequency counts of the relevant conjunctions in the cross-sectional data. Consistent with the longitudinal data, the temporal conjunctions prefer SS. Also consistent is the preference for DS for the circumstantial conjunction *-nuntey* “given that, but” and for the conjunction *-teni* “when/as” denoting initiating event. For the causal conjunctions, *-ese* and *-ekaciko*, the cross-sectional data clearly show a preference for DS. The DS and SS preferences of the conjunctions are consistent across all five child groups and between children and adults, with a few exceptions (e.g., adults’ use of *-teni*), are hard to interpret due to their low token frequencies. Overall, together with the longitudinal data, the present study suggests that the topic continuity features of conjunctive functions develop from the onset of the acquisition of clause chaining.

**TABLE 7:** Topic continuity feature (DS vs. SS) of conjunctions: cross-sectional study.

	**SS preferred**						**DS preferred**									
	**Sequence/manner**		**Simultaneity**		**Interrupted event**		**Circumstance**		**Initiating event**		**Cause**		**Cause**		**Total**	
	***-ese***		**-*myense***		***-taka***		***-nuntey***		***-teni***		***-ese***		***-ekaciko***			
**Age group**	**DS**	**SS**	**DS**	**SS**	**DS**	**SS**	**DS**	**SS**	**DS**	**SS**	**DS**	**SS**	**DS**	**SS**	**DS**	**SS**
3 years	1	9	0	0	1	3	1	0	2	0	10	1	1	0	**16**	13
4 years	2	3	1	4	4	3	6	1	8	0	15	6	10	0	**46**	17
6 years	2	1	0	0	3	4	9	2	0	0	27	7	2	2	**43**	16
8 years	0	11	1	4	1	6	4	2	3	0	25	5	21	0	**55**	28
10 years	2	11	3	5	0	9	2	3	0	0	49	11	4	2	**60**	41
Adults	0	37	0	11	0	9	3	3	0	3	43	25	0	0	46	**88**
Total	7	**72^1^**	5	**24**	9	**34**	**25**	11	**13**	3	**169**	55	**38**	4	266	203

*^1^Boldface refers to the preferred type (DS or SS) for the given conjunctive form/function for all age groups combined and the totals of each age group.*

An interesting difference between children and adults is in the total frequencies of DS and SS used to describe the seven events (see the “total” column in Table 7). In describing the video events, children of all age groups produce more DS than SS conjunctions overall, but the adults show the opposite pattern, producing many more SS (88 tokens) than DS (46 tokens). This was mostly due to the adults producing far more tokens with the “sequential” *-ese* conjunction and the simultaneity *-myense* conjunction than the children. Conversely, the children produced many more causal conjunctions (*-ese, -ekaciko*) with DS preference. We will examine possible sources of this difference in the section “Event Segmentation in Clause Chaining.”

### Referential Ambiguity of Subject Argument

In Korean, subject or object arguments are typically expressed either as full NPs or zero anaphora (see the section “Non-finiteness/Finiteness of the Verb/Predicate of a Medial Clause”). Pronouns are infrequently used. In the present data, there are only 14 instances of pronoun use. All of them are used by 3-, 4-, and 6-year-olds, except for one used by an adult. This confirms that for adult speakers, zero anaphora is the preferred choice over pronouns for anaphoric reference (Kim, 1992).

In Korean, appropriate referential choices/forms would be achieved by (i) using a full NP when introducing an entity for the first time in the discourse or when differentiating the referent from several possible entities mentioned in preceding discourse, and (ii) using zero anaphora when the subject of the current clause has the same reference as the subject of the immediately preceding clause. Example (42a–c) shows appropriate referential choices. The 10-year-old follows the above “rules” and thus communicates the intended referents clearly in each clause: The child uses full NPs to introduce the ball and the bottle in the first clause (42a), then refers to the bottle with a full NP again in the second clause (42b) to disambiguate it from the ball. Then in the final clause (42c), the child uses zero anaphora to refer to the bottle, which is the only entity mentioned in the immediately preceding clause (42b), thus is the only candidate for the subject of *kkayci-ess-eyo* “broke.”


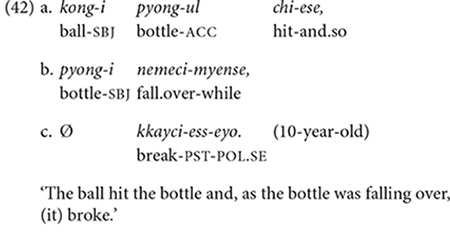


When the speaker does not adhere to the above rules, ambiguity would occur, as in the following examples (43–44). In (43a,b), the 3-year-old, who is describing the ball-bottle event as in (42), introduces the ball rolling (43a), but in the very next clause (43b) states that something broke using zero anaphora. The use of zero anaphora is not appropriate here – and would cause a communication break-down – as the child has never mentioned the entity (=vase) that broke.


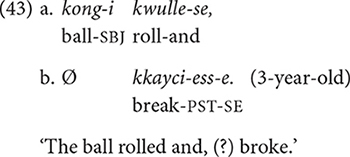


In (44a,b), the child introduces two entities, a ball and a book, in the first clause (44a), but uses zero anaphora in the second clause (44b), not specifying which of the two objects ends up going to the wall. Note that while it is contextually more likely that the book – rather than the ball – propels forward to the wall as a result of it being pushed, in principle, either object could move toward the wall, hence there is potential ambiguity in the listener’s understanding of the event.


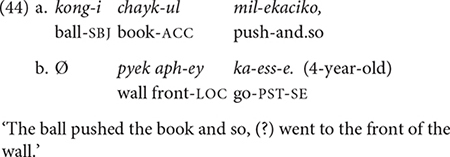


As each event in this elicitation experiment has two entities that move and interact with each other, I examined the extent to which children make appropriate referential choices for the subject argument. Table 8 shows the percentages of ambiguous subject reference in each age group. Three-year-olds clearly have difficulty referring to subjects, as 42.9% of their choices were ambiguous. In large part (42 times out of 48 ambiguity cases), the ambiguity derives from not introducing the intended referent in the description [e.g., (43)].

**TABLE 8:** Frequency and percentages of ambiguous anaphora (zero and pronoun) for subject reference.

**Age group**	**Referential ambiguity type**		**Ambiguous anaphora**	**Clear use of anaphora**	**Full NP**	**Subject reference**	**% Ambiguous anaphora**
						
	**No prior mention of reference**	**Two prior-mentioned entities**	**Sub-total**			**Total**	
3 years	42	6	48	9	55	112	42.86
4 years	21	21	42	9	103	154	27.27
6 years	14	17	31	22	115	168	18.45
8 years	1	22	23	21	134	178	12.92
10 years	0	4	4	42	156	202	1.98
Adults	0	3	3	102	154	259	1.16

Four-year-olds also have difficulty clarifying subject referents (27.3%), but substantially less so than 3-year-olds. Furthermore, 4-year-olds introduce referents with a full NP more often than 3-year-olds do, thus specifying them more clearly. The ambiguity rate drops to 18.5% for 6-year-olds, but they still do not introduce the intended referent half of the time. Eight-year-olds show 12.9% of ambiguity for subject reference, and they are qualitatively different from the younger children in that, except for one instance, they do mention the intended referent in the preceding clauses. But overall, it is at 10 years that the rate of referential ambiguity drops to just 2%.

### Non-finiteness Versus Finiteness of the Verb/Predicate of a Medial Clause

Of all the verbs with conjunctions (children and adult data combined), 12.4% (69 out of 556) have a finite verb form with the past tense marker *-ess*. Similarly to the longitudinal data, the conjunctions that attach to finite verb forms are predominantly *-nuntey* (34 finite out of 36 cases) and *-teni* (11/16), both of which express circumstance, and *-taka* (11/45), which encodes an interrupted action of an agent. Across the age groups, 4-year-olds use finite verbs most often (25%, 18/72) as they frequently use the three conjunctions, *-nuntey*, *-teni*, and *-taka*. The rates of the finite verbs in the other age groups range between 5 and 12%. One may interpret the high frequency of finite verbs in medial clauses among the 4-year-olds as result of their increasing understanding of the disjunctive nature of the three conjunctions.

### Event Segmentation in Clause Chaining

In the present elicitation study, each video event sequence consisted of several sub-events that were temporally and causally sequenced. For example, in the ball-vase event sequence, first, a ball rolls forward and hits a vase which, as a result, tips over and then breaks (see Figure 1, section “Materials and Design”). The sub-events flowed from one to the next without pauses (i.e., no perceptual boundaries). In order to linguistically chain clauses, children first need to cognitively segment connected events into units (Baldwin et al., 2001; Levine et al., 2019), which can then each be expressed as separate clauses and be connected with specification of the semantic relation between them. Given the task of transferring perceptual events to clause chains, we can ask the following questions: which sub-event(s) do children pick out from a series of connected sub-events, and how many sub-events do children connect? To answer these questions, two types of analysis were conducted. In the first analysis, I examined the number of sub-events that participants in each age group expressed for a video event sequence. In the second analysis, I examined the types of sub-event children picked out in an event sequence.

**FIGURE 3:**
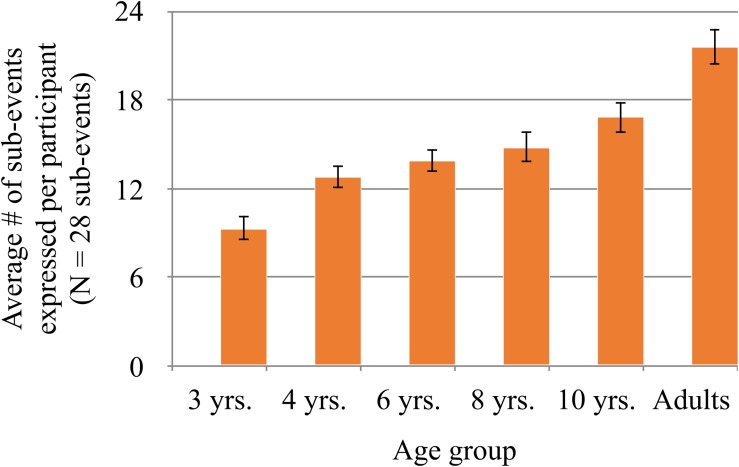
Average number of sub-events expressed per participant by age group, out of the 28 total number of sub-events in the seven video events combined. The number of sub-events expressed by clauses grew as a function of age. See text for statistical results comparing across age groups.


For the first analysis, each video event sequence was broken down into sub-events (Figure 1). The seven video event sequences included 28 sub-events in total, each event sequence having from three to five sub-events (see Supplementary Appendix A). Then, each clause in a description was identified in terms of its corresponding sub-events, and the total number of sub-events expressed for a given video event sequence was counted. Figure 3 shows the average number of sub-events expressed per participant (out of the total 28 sub-events) by age group. Three-year-olds describe about 9.3 sub-events per child. Four-year-olds describe 12.3 sub-events per child. A generalized linear model analysis (with Age as a fixed variable and Sub-event and Participant as random variables) using R statistics, Version 3.5.1 (R Core Team, 2018) shows that the difference between 3- and 4-year-olds is significant (β = 0.866, SE = 0.316, *z* = 2.742, *p* = 0.006). After 4 years, there are no sharp increases from one age group to the next in the children’s data until 10 years. Ten-year-olds are significantly different from 6-year-olds. Also, as can be expected, adults provide significantly more sub-events that 10-year-olds (β = 1.329, SE = 0.460, *z* = 2.887, *p* = 0.004). Some adults provide up to five and six events as follows:

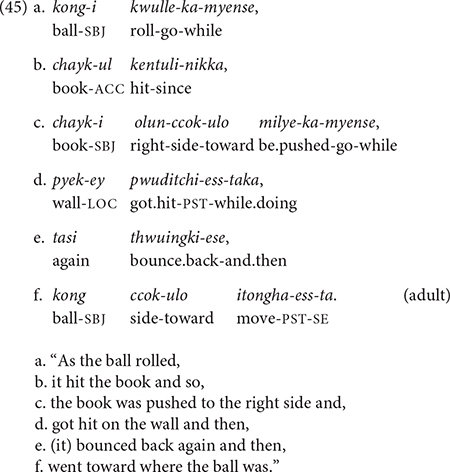


Another way to examine the growth of clause chaining is to compare the frequencies of single vs. multiple clauses in a description. Table 9 shows the number of events that participants in an age group describe in a single clause, two clauses, three–four clauses, or five–six clauses. Out of the total of 84 events per age group (7 events × 12 participants), 3-year-olds describe 53 events in single clauses, each clause referring to one sub-event of a video event. In contrast, almost no adults describe the events with single clauses. The adults describe the events most frequently with three or four clauses, and some with five or six clauses as noted above. Between 4 and 10 years, two-clause descriptions are the most common type. At the same time, the rate of three to four clauses steadily increases from 4 to 10 years. Between 3-year-olds and adults, we observe a substantial decrease in single-clause descriptions at 4 years and again at 10 years. In sum, there are significant increases in the number of sub-events expressed at three age points, 4 years, 10 years, and adults.

**TABLE 9:** Frequency distribution of number of clauses to describe an event by age group.

**Age group**	**1 phrase**	**1 clause**	**2 clauses**	**3–4 clauses**	**5–6 clauses**	**Total**
3 years	4	53	22	5	0	84
4 years	2	24	45	13	0	84
6 years	0	20	49	12	3	84
8 years	0	18	43	22	1	84
10 years	0	5	48	29	2	84
Adults	0	2	25	47	10	84

In the second analysis, I examined the semantic types of sub-event the participants picked out in an event sequence. For this purpose, each sub-event was categorized into one of four event types: (1) object movement (ball rolls/book slides forward), (2) physical contact (ball hits/pushes vase), (3) change of posture (vase tips over), and (4) change of state (vase breaks). Then, each clause in a description was categorized into the corresponding event type, and the number of clauses that expressed a given type was counted per age group. Table 10 shows the proportions of event type expressed by age group.

**TABLE 10:** Proportions of event types expressed by a clause.

**Age group**	**Event type**			
	**Breaking**	**A hitting B**	**Falling**	**Moving**
3 years	0.83^1^	0.36	0.50	0.14
4 years	0.96	0.53	0.65	0.26
6 years	0.92	0.70	0.48	0.24
8 years	0.88	0.80	0.67	0.14
10 years	1.00	0.85	0.67	0.25
Adults	1.00	0.83	0.85	0.63
Average proportion	0.93	0.68	0.64	0.28

*^1^Proportions are calculated by totaling the number of clauses expressing the given event type in each group’s data, and dividing it by the total number of sub-events of the event type presented in the experiment.*

Overall, the type of motion event that participants – both children and adults – describe the most frequently is the perceptually salient change of state, i.e., an object breaking. The next most frequent types are about physical contacts and change of posture, that is, hitting and falling, respectively. The least frequently described sub-event is movement of a single entity, e.g., ball rolling. This overall ranking of frequency is consistent for all age groups. A more detailed look reveals a developmental change in the category of hitting, however (Table 10): There are noticeable increases from 3- to 6-year-olds for the hitting sub-events, such that 3- and 4-year-olds describe the hitting sub-events (0.36 and 0.53 respectively) less often than the falling sub-events (0.5 and 0.65 respectively), whereas children aged 6 years and older show the opposite pattern. Among the four types of events (Table 10), hitting is the only one that involves two entities, one affecting the other. Based on the results, we may hypothesize that younger children have more difficulty expressing two-entity events than one-entity events. This may relate to the earlier finding that younger children have difficulty in referring appropriately to two entities. But, further studies are necessary to test this hypothesis.

One other aspect should be noted. As mentioned earlier, in each group, the least frequently described sub-events are movements of an entity (i.e., the “Moving” event type) that do not involve contact or change of state, e.g., ball rolling. Comparing across the age groups, however, there is a big difference between children and adults: Children of all ages describe such movements <25% of the time, whereas adults express them 63% of the time. This suggests that adults consider a single entity’s movement as an important event to describe, probably because it constitutes an initiating event that explains the ensuing event. For example, in the description (45a–f), (45a) describes the ball’s rolling, which explains how it hit the vase and made it tip over. In the present study, this resulted in adults producing many more clauses with SS compared to children (see the section “Topic Continuity Feature of Conjunctions: Cross-Sectional Study”).

## Summary and Discussion

### Summary

In this paper, I have reported general developmental aspects of clause chaining ability in Korean children, using both naturalistic and elicitation data. The longitudinal naturalistic data have shown that the Korean children in this sample produce clause chaining from at least 2;0. By 2;6, the children connect clauses productively, using many of the language’s major conjunction types that express temporal, causal, conditional, and contrastive relations. For the next year and a half, they develop their clause chaining skills, connecting events that may have temporal and spatial disjunction or interruption. By 4;0, their clause chaining construction is adult-like, in terms of the conjunctive forms, the functions, and their morphological and syntactic features (i.e., finiteness of medial verbs and DS/SS preference of conjunctions). The cross-sectional elicitation data are supported by the findings of the longitudinal data and reveal further developmental aspects. They can be summarized as follows:

(1) Consistent with the longitudinal data, the cross-sectional data show that the major conjunctive types are already in place in 3-year-olds, the youngest age group (Table 6). Then, a significant development occurs from 3 to 4 years, particularly in three areas of clause chaining, conjunction frequency, length of clause chains, and referential choices.
(2) Further developmental milestones occur at 10 years and at adult age. From 4 to 8 years, development from one age group to the next in the three areas are gradual. Overall, the present study shows that clause chaining in Korean develop through adulthood.
(3) Three-year-olds often omit the subject of a clause (i.e., zero anaphora), and its reference is unclear most of the time (Table 8): They often use zero anaphora without having introduced the referent in a prior clause. The ability to appropriately use zero anaphora increases substantially at 4 years and again at 10 years.
(4) Perceptually salient events, such as physical effects of one object on another and change of state or location of an object, are expressed from 3 years. But movements of a single object that do not involve a physical effect or change are not expressed in clause chains until adulthood.

### Discussion

The developmental trajectory from 2 years onward reported in this study suggests that by 4 years of age Korean children have built a solid foundation of the language-specific grammar for constructing clause chains, as they have acquired the major conjunctive form types and their functions. Detailed analyses of the forms and the functions in early years suggest that children acquire them in steps, starting from linking closely related or closely sequenced events/states with the most frequent form (*-ko* “and, and then”) – to expressing event relations that are more complex, including disjunctive events (*-taka* “while doing” and *-teni* “when, as”), events that provide temporal framework (*-ulttay* “when”) and events that happen in parallel (*-myense* “while”). A similar developmental pattern is reported in Slobin’s (1995) work for Turkish children.

What is still lacking in the 4-year-olds, however, is the ability to connect multiple medial clauses in a series, using several different conjunctions within a sentence. Four-year-olds also lack a full control of zero anaphora, albeit much better than 3-year-olds.^10^ It is at 10 years when children use zero anaphora unambiguously most of the time. This suggests that 10-year-olds have a solid grasp of the function of zero anaphora. But adults still differ from 10-year-olds in that they use zero anaphora more extensively than 10-year-olds and do so with clarity. The results suggest that the development of clear anaphoric referencing is gradual and takes time. However, this developmental trajectory is based on experimental data. One needs to examine children’s behavior of anaphoric referencing in diverse discourse genres (e.g., narrative, conversation) to fully understand how Korean children master the system, similar to Clancy’s (1992) on Japanese.

With regard to event segmentation, the data show that while changes of state/location are well picked up on and described, movements of a single object are not expressed in clause chains till adulthood. This finding converges with well-known findings in developmental research that from early on children talk about change of state – as it is perceptually salient – more than on-going event or non-changing state (Antinucci and Miller, 1976; Aksu-Koç, 1998). Thus, children’s early words typically include “broke” or “fell” (Slobin, 1985; Hickmann, 2003), or “gone” denoting disappearance of entity (Gopnik and Meltzoff, 1986). The finding also relates to more recent studies on the development of memory and linguistic expressions for source and goal (e.g., Papafragou, 2010; Lakusta and Carey, 2015; Lakusta et al., 2017): Infants, children, and adults alike, attend more, thus remember better, the goal than the source of an event. For example, having seen an event such as “a car drove from a gazebo into a garage,” participants remember better and describe more often the goal (i.e., the car entering the garage) than the source (i.e., the car leaving the gazebo) (Papafragou, 2010). These findings can be compared to the present finding that children and adults alike describe the end result (e.g., vase falling and breaking) more than the initiating event that involve a single object moving (e.g., ball rolling). On the other hand, it is possible that children in the present study did notice the ball rolling event but were constrained by the number of clauses they can conjoin linguistically. A study that systematically contrasts the two possibilities would provide insight into the question.

While the present study examined the development of clause chaining in Korean in some detail, it has several limitations. First, the study could not systematically investigate the effect of input frequency effect. In this paper, I presented the developmental sequence from a cognitive perspective, but other factors, particularly input frequency, may explain the particular developmental pattern (Lieven, 2010). Second, the longitudinal database is limited in sample size and needs to be expanded to confirm the developmental trajectory reported in the present study. Third, the elicitation data are limited to a very short video-clips of temporally and causally related events. Further experiments can investigate more diverse relations, such as interruptions of events or contrastive events.

Within these limitations, however, the present study has demonstrated – based on both longitudinal case studies and experimental cross-sectional studies – that Korean children acquire major aspects of clause chaining from a young age. It has specified some details of how children develop clause chaining skills and has also laid out important milestones in the development of clause chaining in Korean.

## Data Availability Statement

The datasets generated for this study can be available on request to the corresponding author.

## Ethics Statement

The studies involving human participants were reviewed and approved by the Yeungnam University and the San Diego State University. Written informed consent to participate in this study was provided by the participants’ legal guardian/next of kin.

## Author Contributions

SC organized the database, coded and analyzed all the data, and wrote the manuscript from first draft till the final version.

## Conflict of Interest

The author declares that the research was conducted in the absence of any commercial or financial relationships that could be construed as a potential conflict of interest.

## Footnotes

1 SE *-ta* in spoken conversation has a modal meaning of new information based on perceptual evidence (Choi, 1995, in press).

2 Kim (1992) does not report the remaining nine conjunctive forms.

3 The lower number of conjunctions in Kim’s database compared to the present study is probably due to the fact that Kim’s data are restricted to narratives of a single story, whereas the mother–child interactions in the present study were spontaneous and on a variety of topics.

4 Another type of the V_1_-e V_2_ construction (a subtype of SVC) is AUX/predicate construction (AVC) in which V_2_ functions as an AUX losing its full lexical meaning. In the following example (Suh, 2000, p. 69), V_2_, *po*-, whose lexical meaning is “to see,” serves to be an AUX meaning “try to”:

5 The PI was Dr. Hyeonjin Lee at Yeungnam University in South Korea. I am grateful for her permission to analyze the data collected under her direction.

6 It does not seem to be the case that the children in Dataset 2 overgeneralize already-acquired temporal conjunctive forms (e.g., *-nuntey* “given that”) to include the “initiating event” function of *-teni* “when/as.” An examination of Dataset 2 shows that there are only two cases – medial clauses – in which the four children could have used the conjunction *-teni*.

7 I use the term “listing” (Kim, 1992) as a general term that encompasses simple listings of events that do not have a relationship as well as an “additive” relation that has similar structure with the preceding clause.

8 I thank a reviewer for pointing out that *-nuntey* may express an interactional component in conversation, namely, the speaker’s own perspective (“my-side-telling” to borrow the term from Kim, 2018) that contrasts with a typical expectation or social norm (Park, 1999; Kim, 2018). This subtle relationship between the *-nuntey* and the main clauses may explain young children’s difficulty in using the form appropriately.

9 This experiment was designed by Prof. Maya Hickmann in University of Paris, and I collected the Korean portion of the data. Prof. Hickmann has permitted me to analyze the Korean data for research purposes and for publication.

10 One could argue that 3- and 4-year-olds may rely on the listener’s ability to retrieve the necessary information through contextual cue. For example, in natural conversation, the context is often familiar or set up between the child and the mother, such that even when the child uses zero anaphora ambiguously, the mother would understand. What the present elicitation data has shown is the lack of control in referential choices by 3- and 4-year-olds in a neutral unfamiliar setting.

## Abbreviations

ACC: accusative
AUX: auxiliary verb
COMP: complementizer
CONN: connective
COP: copula
DAT: dative
FUT: future
HON: honorific
HORT: hortative
IMP: imperative
LOC: locative marker
NEG: negative
PASS: passive
POL: polite
PROG: progressive
PRS: present
PST: past
SBJ: subject
SE: sentence-ending
TOP: topic.
