# Supplementary material for: Development of Clause Chaining in Korean
Source: Front Psychol. 2020 Mar 11;11:256. doi: 10.3389/fpsyg.2020.00256 (PMC7078364; doi:10.3389/fpsyg.2020.00256)
Supplement: Supplementary file 1 [file Data_Sheet_1.PDF]

Appendix A

List of video events and their sub-events in the elicitation experiment.

EVENT	Sub-events	# of sub-events
Event 1	A blue ball rolls (1), and hits a red ball (2). As a result, the red ball then rolls forward (3), touches the wall (4), and stops at the wall (5).	5
Event 2	A ball rolls (1), and hits a book (2). As a result, the book moves forward (3), hits a wall (4) and bounces back towards the ball (5)	5
Event 3	A ball rolls (1), and hits a stacked-up dices (2). As a result, the dices fall down (3).	3
Event 4	A ball rolls (1), and hits a wine bottle (2). As a result, the bottle falls down (3), and breaks (4).	4
Event 5	A ball rolls (1), and hits a gift box (2). As a result, the box slides forward (3), and collides with the wall (4).	4
Event 6	A ball rolls (1), and hits several bowling pins (2). As a result, the pins fall down (3)	3
Event 7	A ball rolls (1), and hits a vase (2). As a result, the vase tips over (3) and breaks into pieces (4)	4
**TOTAL**		**28**
